# Bioconversion-Based Postbiotics Enhance Muscle Strength and Modulate Gut Microbiota in Healthy Individuals: A Randomized, Double-Blind, Placebo-Controlled Trial

**DOI:** 10.3390/nu17243937

**Published:** 2025-12-17

**Authors:** Seung Hyeon Jung, Subin Hwang, Kun-Ho Seo, Yongsoon Park, Mi Jung Kim, Hyunsook Kim

**Affiliations:** 1 Department of Food & Nutrition, College of Human Ecology, Hanyang University, 222 Wangsimni-ro, Seongdong-gu, Seoul 04763, Republic of Korea; 2 Center for One Health, Department of Veterinary Public Health, College of Veterinary Medicine, Konkuk University, 120 Neungdong-ro, Gwangjin-gu, Seoul 05029, Republic of Korea; 3 Department of Rehabilitation Medicine, College of Medicine, Hanyang University, 222 Wangsimni-ro, Seongdong-gu, Seoul 04763, Republic of Korea

**Keywords:** postbiotics, *Lentilactobacillus kefiri*, gut microbiome, sarcopenia, irisin

## Abstract

Background: Postbiotics produced by kefir lactic acid bacteria through bioconversion of polyphenol-rich extract and whey protein are emerging as promising modulators of gut microbiota and muscle health. This study investigated whether *Lentilactobacillus kefiri* DH5-derived postbiotics, prepared with *Cucumis melo* L. and whey protein (KP, Kefir lactic acid bacteria-derived postbiotics), improve muscle strength and gut microbiota composition in healthy adults. Methods: In this 12-week, randomized, double-blind, placebo-controlled trial, participants consumed either KP (6 g/day) or placebo. Handgrip strength, circulating biomarkers, and fecal microbiota profiling (using 16S rRNA sequencing) were analyzed. Correlations between microbial taxa and muscle-related biomarkers were assessed. Results: KP supplementation significantly increased dominant-hand grip strength and plasma irisin and reduced IL-1β concentrations after 12 weeks, whereas IGF-1, lean mass, and non-dominant grip strength showed no significant changes. Gut microbiota profiling revealed enrichment of *Bifidobacterium adolescentis*, *Latilactobacillus sakei*, *Lentihominibacter hominis*, *Mediterraneibacter gnavus*, *Streptococcus anginosus* and *Phocaeicola plebeius*, with concomitant reductions in *Lachnospira eligens*, *Roseburia inulinivorans*, *Ruthenibacterium lactatiformans* and *Vescimonas fastidiosa*. Notably, relative abundance of *Faecalibacterium prausnitzii* was positively correlated with plasma irisin concentration. Conclusions: KP supplementation produced a modest within-group improvement in grip strength, potentially through gut–muscle axis modulation involving irisin and anti-inflammation pathways. These preliminary findings suggest that kefir-derived postbiotics may have potential relevance for muscle health.

## 1. Introduction

Sarcopenia, characterized by an age-associated decline in skeletal muscle mass and strength, represents a major public health concern in aging societies, being closely linked with frailty, falls, disability, and reduced quality of life. Emerging clinical evidence indicates that oxidative stress, malnutrition, and dysregulated adipokine and biochemical/hematologic profiles in older adults—such as those observed in hospitalized patients with elevated oxidative burden and impaired nutritional status [1,2]—contribute to accelerated functional decline, underscoring the need for early interventions beginning in midlife to preserve muscle function and maintain long-term metabolic and nutritional resilience. While resistance exercise and adequate protein intake remain cornerstone strategies to mitigate muscle loss, the mechanistic relevance of gut microbial imbalance in the pathogenesis of sarcopenia suggests an important role for the gut–muscle axis [3,4]. A reduction in gut microbial α-diversity and alterations in microbial composition—particularly reducing short chain fatty acid (SCFA)-producing taxa such as *Faecalibacterium prausnitzii*, *Roseburia*, *Ruminococcus*, *Prevotella*, *Slackia*, *Agathobacter*, and *Alloprevotella*—along with an enrichment of potentially pathogenic genera including *Escherichia–Shigella*, *Eggerthella*, and *Collinsella aerofaciens* have been associated with muscle atrophy and sarcopenia severity [5]. These microbes modulate host inflammation, energy metabolism, and mitochondrial function [3], thereby influencing the anabolic–catabolic balance in skeletal muscle. Collectively, these findings underscore gut microbiota modulation as a novel preventive and therapeutic avenue for sarcopenia.

Gut–muscle crosstalk is mediated through multiple mechanisms, including reinforcement of intestinal barrier integrity, attenuation of endotoxin (lipopolysaccharide) translocation, and production of microbial metabolites such as SCFAs, bile acids, and essential amino acids. These bioactive compounds regulate low-grade inflammation, insulin sensitivity, and mitochondrial biogenesis—key processes for muscle maintenance and function [6,7,8].

Dietary strategies that restore eubiosis have therefore been proposed as promising interventions to support muscle health by enhancing SCFA production and nutrient absorption [9,10]. Among these, postbiotics—non-viable microbial cells, metabolites, or structural components—offer unique translational advantages, including stability, safety, and predictable bioactivity. Postbiotics-derived *Akkermansia muciniphila* HB05, *Bifidobacterium breve* BB091109, *Lacticaseibacillus paracasei* MCC1849, and *Lacticaseibacillus paracasei* PS23 have been shown to improve inflammatory status, immune function, gut microbiota composition, and muscle strength in healthy adults and older adults [11,12,13,14]. In our previous study, kefir-derived postbiotics generated through bioconversion of whey protein and polyphenol-rich substrates such as *Cucumis melo* L. peel by *Lentilactobacillus kefiri* DH5 provided synergistic benefits on host metabolism and microbiota remodeling in a hindlimb-immobilized mouse model. These effects may result from the combination of microbial metabolites (e.g., SCFAs, S-layer proteins, glycosidases) and bioconverted polyphenols, which enhance antioxidant and anti-inflammatory capacities, potentially activating IGF-1-mediated anabolic pathways and suppressing proteolytic factors such as *Atrogin-1*, thereby contributing to muscle preservation. Bioconversion, defined as the microbial transformation of organic substrates into bioactive compounds [15], plays a central role in these effects. We have also demonstrated the enhancement of muscle function by extracellular vesicle (EV) postbiotics derived from *L. kefiri* DH5 [16].

However, clinical evidence supporting the muscle-protective effects of postbiotics and their capacity to modulate gut microbiota in humans remains limited. Therefore, the present study aims to investigate the effects of 12-week supplementation with kefir-based postbiotics—derived from bioconversion of whey and *Cucumis melo* L. peel extract—on muscle strength, inflammatory and anabolic biomarkers, and gut microbiota composition in healthy middle-aged subjects through a randomized, double-blind, placebo-controlled clinical trial.

## 2. Methods

### 2.1. Study Design and Participants

A 12-week, randomized, double-blind, placebo-controlled study was conducted at the Hanyang University Hospital in Seoul, South Korea. The study protocol to determine the effect of postbiotics (KP, kefir lactic acid bacteria-derived postbiotics, *L. kefiri* DH5 derived bioconverted product of *Cucumis melo* L. and whey protein) was approved by the Hanyang University Institutional Review Board (HYUIRB-2023-07-059) and carried out in accordance with the Declaration of Helsinki and registered with ClinicalTrial.gov (NCT06230302). Written informed consent was provided from all participants before implementing the study. Participants were required to follow inclusion and exclusion criteria. Inclusion criteria were adults aged 40–65 years with total Charlson Comorbidity Index (CCI) scores = 0 and skeletal muscle mass < 110%. We excluded participants with chronic medical conditions because comorbidities such as diabetes or cardiovascular disease can independently influence inflammation, physical activity levels, and gut microbiota composition. Restricting the cohort to a CCI = 0 ensured a more homogeneous population and minimized potential confounding, allowing a clearer evaluation of the postbiotic intervention. Skeletal muscle mass < 110% refers to the In Body SMM index, where “<110%” indicates skeletal muscle mass below 110% of age/sex-matched reference values. This range identifies individuals at risk for low muscle mass but without diagnosed sarcopenia. Exclusion criteria were as follows: (i) smoker, vegetarianism, having hypersensitivity to oriental melon, whey or probiotics; (ii) AST or ALT level > 1.5 times the normal upper limit, serum creatinine > 1.4 mg/dL, fasting blood sugar > 126 mg/dL, 2+ or more protein in a urine test, BMI > 30 kg/m^2^; (iii) having uncontrolled acute or chronic diseases; (iv) history of gastrointestinal surgery (excluding appendectomy); (v) participation in other clinical studies, diet or exercise programs within 3 months; (vi) taking laxatives or antidiarrheal, psychiatric medication before screening; (vii) taking probiotics, prebiotics or other test-related products within 1 month before screening, (viii) taking antibiotics within 2 months before screening; (ix) taking protein supplements regularly for more than 3 months within 6 months before screening; (x) adjusting diet for disease management purposes; (xi) currently performing or planning to perform regular resistance exercises; and (xii) having a plan for weight loss within 6 months after screening.

**Figure 1:**
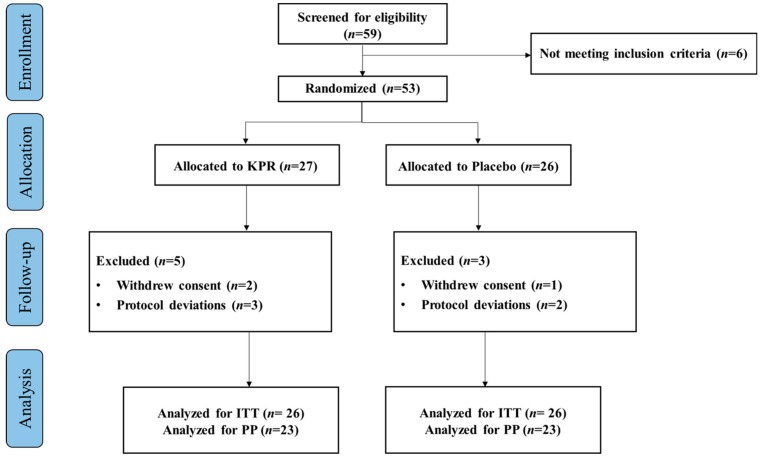
Flow chart diagram of participant enrollment, randomization, allocation, and analysis.

A total of 59 participants were screened for eligibility and 53 participants as part of ITT population, randomly assigned to the KP (*n* = 27) or placebo groups (*n* = 26). Three participants withdrew their study consent, and five participants violated the protocol deviation. Altogether 45 participants completed the study (*n* = 22 KP; *n* = 23 placebo, PP), with >80% compliance. The study flowchart is depicted in (Figure 1).

The study consisted of screening and baseline within 4 weeks, and two follow up assessment visits (week 6 and week 12). During the screening visit, information regarding demographics, medical and medication history, alcohol intake, smoking status and exercise levels was collected. Within four weeks of the screening, eligible participants were randomized in a 1:1 ratio to KP or placebo group using permuted block randomization. A researcher assigned the participants identity code, and the participants were blinded to the code during intervention. Blinding codes were managed by a third-party individual who was not related to this study. Code breaking was performed after statistical analysis was completed. The participants were required to take a total of 6 g of either KP or placebo for 12 weeks. The remaining samples were collected at week 6 and week 12 to evaluate supplement compliance. KP dose was determined from previous study, which showed that 6 g of KP supplementation for a 60 kg human significantly increased muscle mass and improved muscle strength. Six grams of KP provides approximately 18.8 kcal, 2.6 g of protein, 3.4 g of carbohydrates, and 0 g of fat, insufficient for meaningfully influencing total daily caloric intake. During the intervention, the participants were asked not to consume any other probiotics, antibiotics, laxatives and antidiarrheal medication.

### 2.2. Outcomes

#### 2.2.1. Blood/Stool Sample Collection

Blood samples were collected after overnight fasting at baseline and week 12 for biochemical analysis. Blood was centrifuged at 3000× *g* rpm for 10 min at 4 °C, and blood serum was stored at −80 °C until further analyses. For the collection of stool samples, the participants were provided with stool tube with a scoop inside the tube and were instructed to gather stool sample at baseline and week 12. The samples were frozen at −80 °C until further analysis.

#### 2.2.2. Primary Outcome

Hand grip strength was measured using handheld a dynamometer T.K.K.5401 (Takei, Tokyo, Japan) at baseline and week 12. The value for grip strength was recorded three successive trials and the average value was used. The values for BMC, BMD, fat mass, lean mass, Z-score, and T-score were measured by Horizon^®^ DXA System (Hologic Inc., Marlborough, MA, USA) at baseline and week 12. To measure body composition, Inbody 120 (Biospace, Seoul, Republic of Korea) was used at baseline. Hematologic tests were conducted using an XN-10 analyzer (Sysmex, Kobe, Japan), blood chemistry tests were measured using an AU 5800 automated analyzer (Beckman Coulter Inc., Brea, CA, USA), and urine analysis was conducted using a Cobas 6500 automated urine chemistry analyzer (Roche Diagnostic, Mannheim, Germany). Blood pressure and pulse rate were measured using an Omron HBP-9030 (Omron Healthcare, Kyoto, Japan) and body temperature was measured using a Tommy original HET-1000 (HuBDIC, Anyang, Republic of Korea) at each visit. Dietary intake was estimated by one-day dietary record at baseline and week 12 and data were analyzed using CAN-pro 6.0 (Korean Nutrition Society, Seoul, Republic of Korea). Physical activity level was assessed by a Global Physical Activity Questionnaire (GPAQ). The metabolic equivalent task (MET) was estimated using GPAQ data, representing the relative ratio of working metabolic rate to resting metabolic rate. Adverse events were monitored throughout the study.

#### 2.2.3. Secondary Outcome

IL-1β and IL-10 were measured by Cytokine 4-plex A Kit (Quantikine, Minneapolis, MA, USA). IL-2, IGF-1, Myostatin, and Irisin were measured using Human IL-2 ELISA kit (Quantikine, Minneapolis, MA, USA), Human IGF-1/IGF-1 ELISA kit (Quantikine, Minneapolis, MA, USA), Human GDF-8/Myostatin ELISA kit (Quantikine, Minneapolis, MA, USA) and Human irisin ELISA kit (MyBioSource, San Diego, CA, USA), respectively. Metagenomic DNA was extracted from stool samples. The 16S V3-V4 regions of the rRNA genes were amplified using the 341F-805R primer pair and sequenced on an Illumina HiSeq 2000 (Illumina, Inc., San Diego, CA, USA). After sequencing, adapter and primer sequences from the raw data were removed and paired-end reads were trimmed to 250 bp and 200 bp, both using Cutadapt (v3.2). To generate Amplicon Sequence Variants (ASVs), error-correction, denoising, and merging procedures were performed on the reads using DADA2 (v1.18.0, Nashville, TN, USA). Reads with expected error rates of ≥2 were excluded, and paired-end reads were merged. Chimeric sequences were eliminated using the remove Bimera De novo function in consensus mode in DADA2. ASVs shorter than 350 bp were excluded using R (v4.0.3) and normalization was performed for microbial community comparison using QIIME (v1.9). To ensure comparability between the samples, subsampling was performed using the sample with the lowest read count. Taxonomic assignment of each ASV was conducted using Bayesian classifier (DADA2_v1.18.0, confidence value: 50) algorithms against the reference database such as NCBI 16S. Alpha diversity (Shannon, Gini-Simpson, and PD whole tree indices) was computed using mafft (v7.475) and FastTreeMP (v2.1.10) and beta diversity (Bray–Curtis, Weighted UniFrac, and Unweighted UniFrac distance) was visualized based on PCoA.

### 2.3. Statistical Analysis

The sample size was estimated using G Power 3.1. An effect size of lean mass gain of 3.3 ± 1.5 kg (mean ± SD) in the experimental group and 1.8 ± 1.6 kg in the placebo group was used for the calculation, based on previous study [17]. With a significant level of 5% and power (1 − b) = 0.80, these assumptions necessitated 24 participants per arm (total of 48).

Intention-to-treat (ITT) analysis and per-protocol (PP) analysis were conducted for primary outcome. In the ITT analysis, missing data were imputed using last observation carried forward (LOCF) method. PP analyses include participants who completed the study with a more than 80% compliance rate. Secondary outcomes were analyzed using PP analysis only. Eight participants (*n* = 5 KP; *n* = 3 placebo) excluded in the PP analysis did not provide adequate stool samples or consumed less than 80% of the study product. Normality tests were performed using the Shapiro–Wilk test. Variables were expressed as mean ± standard deviation (SD) and counts (percentage). Baseline characteristics were assessed using independent *t*-test. If the variables were normally distributed, an independent *t*-test was used to compare the changes between KP and placebo from baseline to week 12, and if the variables were not normal, Mann–Whitney U-test was performed. Likewise, within-group comparisons were analyzed using paired *t*-test if the variables were normal, and Wilcoxon test was used if the variables were not normal. Data representing < 0.01% of the relative abundance were excluded from the microbial profile analysis. Spearman’s rank correlation was used to analyze the association between muscle function profiles and microbial profiles. Statistical analysis was performed using IBM SPSS statistics 28.0 (SPSS Inc., Chicago, IL, USA) and values of *p* < 0.05 were regarded as statistically significant.

## 3. Results

### 3.1. Primary Outcome: Dominant Hand Grip Strength Significantly Increased in the KP Group After 12 Weeks of Intervention in Both ITT and PP Analysis

Baseline characteristics including age, sex, height, weight and BMI were comparable between the KP and placebo groups (Table 1). There was no reported adverse event related to the intervention during study.

Physical activity levels (MET-min/week) and dietary intake variables (energy, carbohydrate, fat, protein, and fiber) did not differ significantly between groups at baseline. Across the 12-week intervention, neither group exhibited significant within-group or between-group changes in physical activity or nutrient intake (Supplementary Table S1). Although the KP group exhibited a modest numerical increase in caloric intake, this change was not statistically significant (*p* = 0.057–0.080). To account for this potential source of confounding, we performed an ANCOVA using the change in dominant-hand grip strength as the dependent variable, treatment group as the fixed factor, and baseline energy intake and change in energy intake (baseline → week 12) as covariates. Adjustment for energy intake did not alter the primary inference, as the between-group difference in grip-strength change remained non-significant, consistent with our original ITT and PP analyses.

In both ITT and PP analysis, between-group comparisons did not reveal a statistically significant difference in dominant-hand grip strength after 12 weeks (ITT: *p* = 0.498; PP: *p* = 0.36; Table 2). However, within-group analyses showed a significant increase in dominant-hand grip strength in the KP group (ITT: *p* = 0.01; PP: *p* = 0.03), whereas the placebo group showed no significant change. No significant between-group differences were observed for any body composition variables, including fat mass, lean mass, bone mineral content, or bone mineral density (Table 2). Hematologic parameters and vital signs also remained comparable between groups at baseline and week 12 (Supplementary Tables S2 and S3).

### 3.2. Secondary Outcome: PP Analysis

#### 3.2.1. Inflammatory and Muscle Biomarkers

Between-group comparisons of inflammatory biomarkers did not reach statistical significance. Within-group analyses showed that IL-1β decreased significantly only in the KP group (*p* = 0.011), while IL-10 increased significantly only in the placebo group (*p* = 0.001). Myostatin levels decreased in both groups (*p* < 0.05) (Table 3).

#### 3.2.2. Microbial Profiling

**Figure 2:**
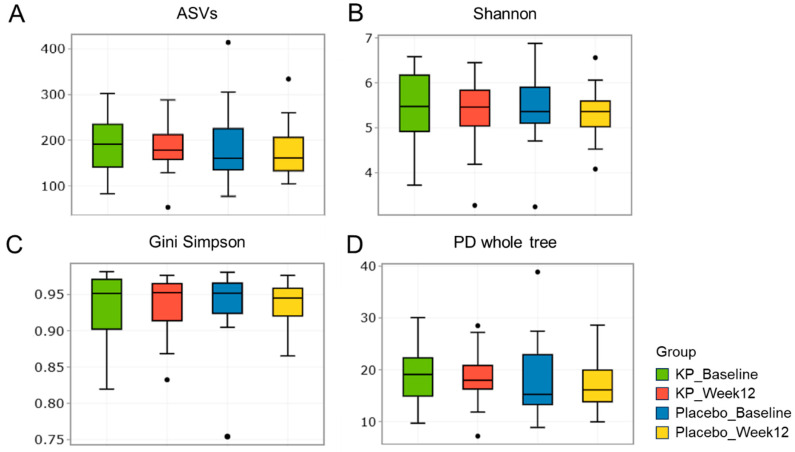
α-diversity of gut microbiota in the KP and placebo groups at baseline and week 12. (**A**–**D**) Boxplots display α-diversity indices, including (**A**) ASV richness, (**B**) Shannon diversity, (**C**) Gini Simpson, and (**D**) PD whole tree, for the KP group at baseline (green) and week 12 (red), and the placebo group at baseline (blue) and week 12 (yellow).

**Figure 3:**
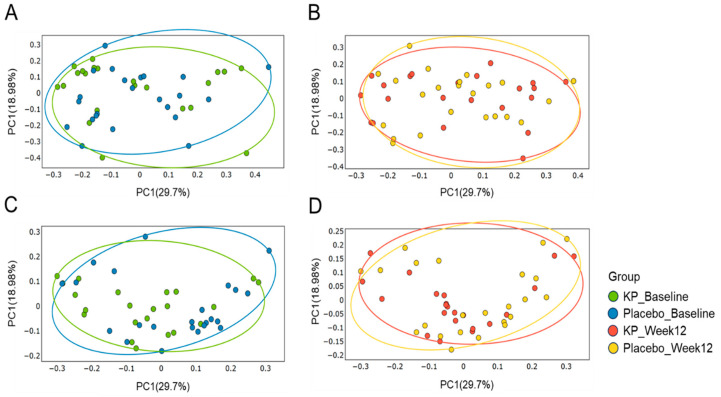
Principal Coordinate Analysis (PCoA) of gut microbiota β-diversity in the KP and placebo groups at baseline and week 12. Weighted UniFrac–based PCoA plots are shown for baseline (**A**) and week 12 (**B**), and unweighted UniFrac–based plots are shown for baseline (**C**) and week 12 (**D**). Colors represent the KP group at baseline (green) and week 12 (orange), and the placebo group at baseline (blue) and week 12 (yellow).

**Figure 4:**
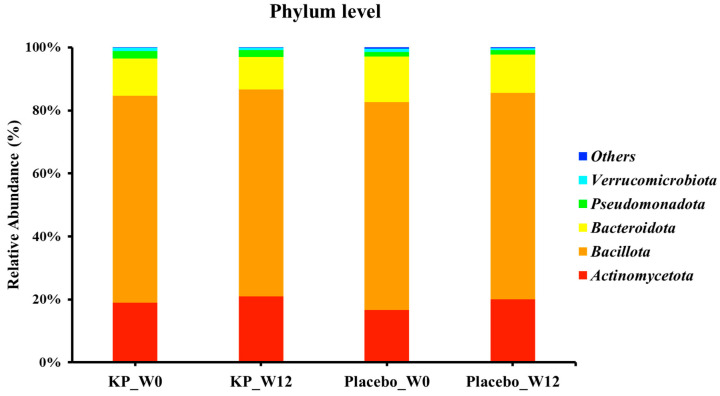
Relative abundance of gut microbiota at the phylum level in the KP and placebo groups at baseline and week 12. KP at baseline (KP_W0), KP at week 12 (KP_W12), placebo at baseline (Placebo_W0), and placebo at week 12 (Placebo_W12).

**Figure 5:**
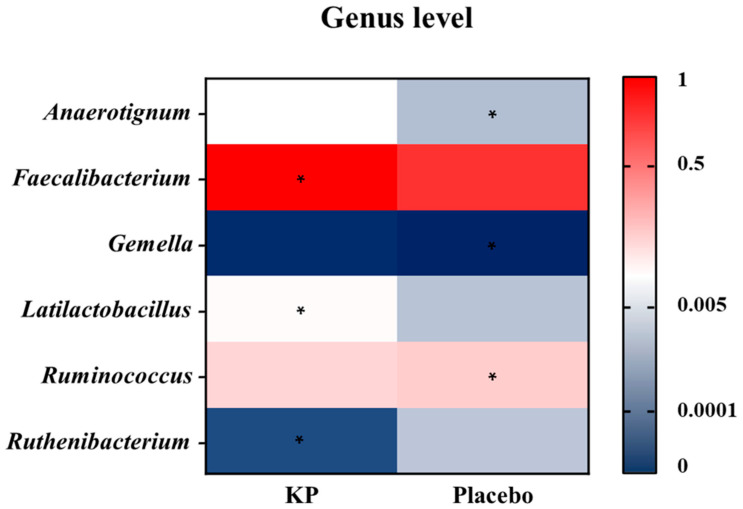
Genus-level heatmap showing changes in relative abundance after 12 weeks of KP or placebo supplementation. Colors represent scaled relative abundance values ranging from low (blue) to high (red). Asterisks (*) indicate genera showing a statistically significant within-group change from baseline to week 12 (*p* < 0.05).

There were no significant between-group differences in α-diversity indices (ASVs, Shannon, Gini-Simpson, and PD whole tree index) for at baseline or week 12 (Figure 2A–D). β-diversity analyses (Unweighted UniFrac and Weighted UniFrac) showed a compositional shift of KP group to placebo group at week 12 compared with baseline (Figure 3A–D). At the phylum level, no significant change was observed following KP supplementation (Figure 4). At the genus level, within-group analyses showed that the KP group exhibited significantly increased relative abundance of *Latilactobacillus* (*p* < 0.05) and *Faecalibacterium* (*p* < 0.05) and decreased relative abundance of *Ruthenibacterium* (*p* < 0.05) after 12 weeks (Figure 5). At the species level, within-group comparisons exhibited significant increases (*p* < 0.05) in the abundance of *Bifidobacterium adolescentis*, *Latilactobacillus sakei*, *Lentihominibacter hominis*, *Streptococcus anginosus*, and *Phocaeicola plebeius*, while significant decreases (*p* < 0.05) in the abundance of *Lachnospira eligens*, *Roseburia inulinivorans*, *Ruthenibacterium lactatiformans*, and *Vescimonas fastidiosa* after 12 weeks of intervention (Table 4).

To minimize noise arising from extremely low-abundance taxa, features with relative abundance below 0.01% were excluded prior to analysis. All taxa highlighted in the present Results—including *Bifidobacterium adolescentis*, *Latilactobacillus sakei*, and *Phocaeicola plebeius*—were all above this filtering threshold in our dataset. Although the observed compositional shifts were modest, these taxa demonstrated consistent patterns across multiple participants, supporting their relevance within the context of the intervention.

#### 3.2.3. Correlation Between Muscle Function Profile and Microbial Profile

Correlations between gut microbiome changes and muscle or inflammation-related markers are shown in Table 5. Dominant grip strength had a positive correlation with *Anaerobutyricum soehngenii* (γ = 0.69; *p* = 0.024), but a negative correlation with *Hominisplanchenecus faecis* (γ = −0.68; *p* = 0.028). IL-1β had a positive correlation with *Coprococcus catus* (γ = 0.72; *p* = 0.013) and *Evtepia gabavorous* (γ = 0.67; *p* = 0.039). Meanwhile, IL-2 had a negative correlation with *Coprococcus comes* (γ = −0.70; *p* = 0.022) and *Enterobacter cloacae* (γ = −0.80; *p* = 0.030). Positive associations were observed between myostatin and *Dorea phocaeensis* (γ = 0.72; *p* = 0.038). Irisin demonstrated a positive association with *Faecalibacillus intestinalis* (γ = 0.66; *p* = 0.045) and a negative correlation with *Turicibacter bilis* (γ = −0.68; *p* = 0.032).

## 4. Discussion

This 12-week, randomized, double-blind, placebo-controlled clinical trial provides the first clinical evidence that daily supplementation with *Lactobacillus kefiri* DH5-derived postbiotics—produced through the bioconversion of whey protein and *Cucumis melo* L. extract-led to significant improvements in dominant-hand grip strength, gut microbiota, plasma irisin, and interleukin-1β (IL-1β). Other anabolic or functional indicators—including IGF-1, lean mass, and non-dominant grip strength—did not change in healthy middle-aged adults. These findings suggest that the observed functional benefits are mediated through modest, targeted physiological responses along the gut–muscle axis, rather than broad activation of systemic anabolic pathways. Although a small numerical increase in energy intake was observed in the KP group, this change was not statistically significant, and adjustment for baseline energy intake and changes in energy intake did not modify the intervention effect on grip strength. Thus, the observed functional improvements are unlikely to be attributable to dietary differences between groups.

Irisin, a cleaved form of fibronectin type III domain-containing protein 5 (FNDC5), is a well-characterized myokine secreted by skeletal muscle in response to exercise [18]. It promotes mitochondrial biogenesis and oxidative metabolism via activation of the AMPK–PGC-1α pathway, stimulates myogenic differentiation, and exerts anti-inflammatory effects by suppressing NF-κB signaling [19,20,21,22]. The elevation in plasma irisin observed in this study may therefore reflect enhanced muscle metabolic activity stimulated by bioactive compounds generated during *L. kefiri* fermentation, such as peptides, polyphenol metabolites, short-chain fatty acids (SCFAs), and microbial extracellular vesicle (EV). Although these metabolites have been shown to modulate myokine secretion and mitochondrial remodeling even in the absence of structured physical exercise, such mechanisms remain speculative in the present study and require further mechanistic verification.

The reduction in IL-1β further supports the anti-inflammatory potential of kefir-derived postbiotics. IL-1β is a key pro-inflammatory cytokine implicated in muscle wasting through its activation of proteasomal degradation pathways and suppression of satellite-cell function [23,24]. Postbiotic-derived metabolites and EVs from *L. kefiri* are known to downregulate IL-1β production by inhibiting NF-κB and Toll-like receptor signaling [25,26]. The concurrent increase in irisin and decrease in IL-1β observed in this study thus suggest a coordinated mechanism linking metabolic activation and inflammatory suppression. Previous reports indicate that irisin can inhibit NLRP3 inflammasome activation and reduce IL-1β expression [27], implying a reciprocal feedback loop between myokine signaling and immune regulation.

Microbiome profiling revealed that KP supplementation selectively enriched anti-inflammatory and SCFA-producing beneficial genera such as *Latilactobacillus*, and *Faecalibacillus*, while reducing potentially pro-inflammatory taxa including *Ruthenibacterium*. The abundance of *Faecalibacillus* has been shown to be significantly decreased in aged individuals with sarcopenia [28,29] and to negatively correlated with grip strength and body mass index (BMI) in healthy women [30]. In contrast, *Ruthenibacterium* abundance was declined in myostatin-inactivated sheep [31], suggesting a possible link between this genus and muscle catabolism. In particular, the increased abundance of SCFA-producing gut bacteria—*Bifidobacterium adolescentis*, *Latilactobacillus sakei*, *Faecalibacillus intestinalis*, and *Anaerobutyricum soehngenii*—was positively associated with both grip strength and circulating irisin concentrations, supporting the hypothesis that microbial metabolites mediate systemic myokine signaling and exert anti-inflammatory effects. Conversely, the relative abundance of *Lachnospira eligens*, *Roseburia inulinivorans*, and *Vescimonas fastidiosa* markedly decreased following supplementation. *Lachnospira eligens* abundance has been negatively associated with protein synthesis in individuals with osteoporosis [32,33], and *Roseburia inulinivorans* plays a role in carbohydrate substrate utilization [34,35]; thus, its reduction may reflect a metabolic shift toward protein substrate utilization during postbiotic fermentation.

Correlation analyses indicated that inflammatory biomarker (IL-1β) was positively associated with *Evtepia gabavorous*—a gamma-aminobutyric acid-metabolizing bacterium linked to IL-1β production [36]—whereas anti-inflammatory cytokine IL-2 showed a significant negative correlation with *Coprococcus comes*, known for its role in inflammatory cytokine production [37]. Collectively, these findings suggest that kefir-derived postbiotics may cultivate a gut microbial ecosystem that supports muscle homeostasis and maintains an anti-inflammatory milieu, potentially contributing to improved muscle performance and metabolic resilience. Although dietary intake did not significantly differ between groups, subtle unmeasured variations in habitual diet may still influence microbiome composition, and this possibility cannot be completely excluded.

These findings are consistent with our previous preclinical study in hindlimb-immobilized mice [38], in which kefir-derived postbiotics improved muscle mass and strength, upregulated myogenic markers such as *MyoD* expression, and enhanced α-diversity of the gut microbiota and the abundance of taxa associated with protein synthesis and butyrate production. In the present human trial, although overall α-diversity did not significantly change, genus- and species-level analyses revealed targeted microbiome modulation with functional relevance.

It is important to note that taxa with relative abundances below 0.01% were removed to avoid inflation of analytical noise typical of extremely low-abundance ASVs. All taxa discussed in the present study were above this threshold and demonstrated reproducible patterns across individuals. Nevertheless, the changes observed at the genus and species levels were generally modest, and the study’s limited sample size restricts the strength of mechanistic inferences. Accordingly, the associations between KP supplementation, specific microbial taxa, and circulating biomarkers should be interpreted as hypothesis-generating rather than conclusive evidence of direct microbial effects.

Interestingly, KP supplementation did not alter plasma IGF-1 concentrations. This observation suggests that the functional benefits of the intervention were primarily mediated through peripheral, muscle-specific pathways rather than systemic endocrine responses. Irisin has been known to reflect enhanced mitochondrial biogenesis and metabolic activity within myofibers. It responds rapidly to local metabolic stimuli, including improved energy utilization and reduced inflammation, both of which are strongly influenced by gut-derived metabolites [5,39,40]. In contrast, circulating IGF-1 is largely synthesized in the liver under growth-hormone regulation and exhibits slower, more variable responses to nutritional and hormonal cues. Its concentration is also known to decline with age and to show limited short-term responsiveness in middle-aged or postmenopausal adults due to reduced growth hormone sensitivity [41]. Therefore, even when muscle metabolism and contractile function improve, systemic IGF-1 levels may remain stable. Moreover, several studies have reported similar findings—enhanced muscle strength and elevated irisin without concurrent changes in circulating IGF-1 [42,43,44]—indicating that local, autocrine/paracrine IGF-1 signaling within skeletal muscle (mIGF-1) may contribute to tissue repair and hypertrophic signaling without affecting circulating concentrations. These results imply that KP may enhance muscle function primarily through modulation of the gut–muscle axis, [45], including the production of short-chain fatty acids (SCFAs) and phenolic metabolites that improve mitochondrial activity and suppress inflammation [46]. These peripheral, metabolism-oriented adaptations appear sufficient to improve muscle strength and energy efficiency, even in the absence of measurable increases in circulating IGF-1. Collectively, this evidence supports the emerging view that postbiotics exert their beneficial effects on skeletal muscle via metabolic, anti-inflammatory, and gut-mediated pathways, rather than through the traditional systemic GH–IGF-1 endocrine mechanism.

Because physical activity levels and dietary intake remained stable over the 12-week intervention and did not differ between the KP and placebo groups, these factors are unlikely to have confounded the observed improvements in dominant-hand grip strength and inflammatory markers. Although caloric intake increased slightly in the KP group, the changes were not statistically significant and did not differ between groups. This minor numerical increase is attributable to natural day-to-day variation rather than the intervention. The absence of significant between-group differences in MET levels or nutrient intake supports the interpretation that the observed effects are attributable to the KP postbiotic intervention rather than changes in lifestyle behaviors.

Interestingly, these functional benefits were achieved with a low daily dose (6 g) of postbiotic powder, substantially lower than conventional whey protein interventions (20–40 g per serving). Whereas conventional whey supplementation depends on amino acid or leucine thresholds to stimulate muscle protein synthesis, the effects observed here likely result from immune–metabolic and microbiome-mediated mechanisms rather than direct amino acid provision. This distinction underscores the potential of postbiotics as exercise-mimetic functional foods, capable of enhancing muscle function and reducing inflammation without requiring high protein intake or resistance training. Such an approach may be particularly beneficial for individuals with low appetite, gastrointestinal intolerance, or limited exercise capacity.

In summary, the current findings indicate that kefir-derived postbiotics produced through *L. kefiri* DH5 fermentation of whey protein and *Cucumis melo* extract improved muscle strength, elevated circulating irisin, and lowered IL-1β levels in healthy adults. These concurrent anabolic and anti-inflammatory responses suggest that the postbiotic may offer a modest, targeted benefits as an exercise-mimetic modulator of the gut–muscle axis, promoting muscle health even in the absence of structured training. The selective enrichment of SCFA-producing taxa and associated microbial signaling pathways further implies that gut-derived metabolites and EVs play a central role in mediating these physiological effects. However, this study demonstrated modest within-group improvements but no significant between-group differences. Therefore, interpretation of efficacy should remain cautious and considered as hypothesis-generating, given the limited sample size and modest effect sizes.

In conclusion, these results highlight kefir-derived postbiotics as a promising dietary strategy for maintaining muscle function and preventing sarcopenia through integrative microbiome–myokine–immune interactions. Future studies integrating metagenomic, metabolomic, and muscle-biopsy analyses are warranted to clarify the molecular mechanisms linking gut microbial metabolites, extracellular vesicle communication, and myokine regulation, ultimately guiding the development of next-generation postbiotic interventions for muscle and metabolic health.

## Figures and Tables

**Table 1:** Baseline characteristics.

	KP	Placebo	
**Intention to treat**	(*n* = 27)	(*n* = 26)	*p*-value
Age (yrs)	53.70 ± 7.45	55.04 ± 8.83	0.465
Female (%)	25 (93)	24 (92)	
Height (cm)	158.50 ± 5.66	159.54 ± 6.20	0.525
Weight (kg)	59.76 ± 9.91	59.93 ± 8.18	0.946
BMI (kg/m^2^)	23.70 ± 3.12	23.52 ± 2.68	0.822
**Per protocol**	(*n* = 22)	(*n* = 23)	
Age (yrs)	52.55 ± 6.46	55.74 ± 8.96	0.179
Female (%)	20 (91)	21 (91)	
Height (cm)	159.59 ± 5.47	159.65 ± 6.53	0.973
Weight (kg)	60.70 ± 9.35	60.52 ± 8.27	0.946
BMI (kg/m^2^)	23.77 ± 2.97	23.72 ± 2.70	0.948

Values are mean ± SD or number (percentage). KP, Kefir postbiotics; BMI, body mass index. *p*-values were evaluated using Mann–Whitney U-test or independent *t*-test.

**Table 2:** Anthropometric measurements.

	KP		*p*-Value ^(1)^	Placebo		*p*-Value ^(1)^	*p*-Value ^(2)^
	**Baseline**	**Week 12**		**Baseline**	**Week 12**		
**Intention to treat**	(*n* = 27)			(*n* = 26)			
Grip strength							
Dominant hand (kg)	19.80 ± 7.09	21.42 ± 6.77	0.012 *	19.77 ± 5.81	20.84 ± 5.22	0.128	0.498
Non-dominant hand (kg)	19.03 ± 8.12	18.65 ± 7.47	0.475	19.39 ± 5.61	20.57 ± 5.46	0.252	0.128
BMC (kg)	1.92 ± 0.31	1.90 ± 0.27	0.379	1.91 ± 0.31	1.91 ± 0.30	0.644	0.541
BMD (g/cm^2^)	1.04 ± 0.09	1.06 ± 0.14	0.582	1.04 ± 0.09	1.04 ± 0.09	0.414	0.343
Fat (kg)	20.88 ± 6.03	21.08 ± 5.72	0.638	20.12 ± 6.78	19.89 ± 6.41	0.861	0.749
Lean (kg)	36.32 ± 5.84	35.86 ± 5.99	0.276	34.98 ± 8.15	36.34 ± 6.21	0.757	0.403
T-score	−0.90 ± 1.11	−0.09 ± 1.14	0.888	−0.94 ± 1.08	−0.98 ± 1.04	0.390	0.626
Z-score	−0.59 ± 0.88	−0.58 ± 0.89	0.787	−0.51 ± 0.80	−0.49 ± 0.79	0.697	0.867
**Per protocol**	(*n* = 22)			(*n* = 23)			
Grip strength							
Dominant hand (kg)	19.55 ± 7.56	21.24 ± 7.32	0.025 *	19.83 ± 5.87	20.90 ± 5.01	0.204	0.363
Non-dominant hand (kg)	19.08 ± 8.94	18.67 ± 8.13	0.531	19.14 ± 5.77	20.22 ± 5.70	0.286	0.251
BMC (kg)	1.95 ± 0.32	1.93 ± 0.27	0.327	1.92 ± 0.33	1.91± 0.32	0.312	0.569
BMD (g/cm^2^)	1.05 ± 0.09	1.08 ± 0.15	0.614	1.05 ± 0.09	1.08 ± 0.15	0.224	0.198
Fat (kg)	20.76 ± 6.31	21.07 ± 5.71	0.570	20.76 ± 6.31	20.51 ± 7.12	0.815	0.577
Lean (kg)	36.95 ± 5.97	36.47 ± 6.25	0.306	36.95 ± 5.97	36.47 ± 6.25	0.738	0.454
T-score	−0.80 ± 1.17	−0.82 ± 1.17	0.761	−0.80 ± 1.17	−0.82 ± 1.17	0.227	0.555
Z-score	−0.52 ± 0.92	−0.51 ± 0.92	0.844	−0.52 ± 0.92	−0.51 ± 0.92	0.895	0.996

Values are mean ± SD. DEXA, dual-energy X-ray absorptiometry; BMC, bone mineral content; BMD, bone mineral density. ^(1)^ *p*-values for comparison between baseline and week 12 were evaluated using Wilcoxon test or paired *t*-test. ^(2)^ *p*-values for changes between KP and placebo groups from baseline to week 12 were evaluated using Mann–Whitney U-test or independent *t*-test. * *p* < 0.05.

**Table 3:** Cytokine parameters and muscle biomarkers.

	KP (*n* = 22)		*p*-Value ^(1)^	Placebo (*n* = 23)		*p*-Value ^(1)^	*p*-Value ^(2)^
	**Baseline**	**Week 12**		**Baseline**	**Week 12**		
**Per protocol**							
IL-1β (pg/mL)	0.02 ± 0.02	0.01 ± 0.00	0.011 *	0.02 ± 0.01	0.02 ± 0.03	0.869	0.054
IL-10 (pg/mL)	1.42 ± 0.70	1.79 ± 0.87	0.108	1.10 ± 0.65	2.27 ± 2.20	0.001 *	0.302
IL-2 (pg/mL)	25.18 ± 22.00	19.95 ± 20.87	0.398	25.73 ± 36.69	20.54 ± 20.12	0.573	0.909
IGF-1 (ng/mL)	78.00 ± 32.25	71.85 ± 30.16	0.783	78.23 ± 22.94	75.56 ± 30.43	0.670	0.937
Irisin (ng/mL)	21.20 ± 9.86	28.11 ± 14.29	0.027 *	27.39 ± 17.31	35.38 ± 22.34	0.119	0.854
Myostatin (ng/mL)	4.07 ± 3.57	2.51 ± 2.39	0.001 *	4.69 ± 3.74	2.85 ± 2.54	0.001 *	0.340

Values are mean ± SD. KP, kefir postbiotics; IL-1β, interleukin-1 beta; IL-10, interleukin-10; IL-2, interleukin-2; IGF-1, Insulin-like growth factor 1. ^(1)^ *p*-values for comparison between baseline and week 12 were evaluated using Wilcoxon test or paired *t*-test. ^(2)^ *p*-values for changes between KP and placebo groups from baseline to week 12 were evaluated using Mann–Whitney U-test or independent *t*-test. * *p* < 0.05.

**Table 4:** Changes at genus–species level.

At Phylum–Genus–Species	KP (*n* =22)		*p*-Value ^(1)^	Placebo (*n* =23)		*p*-Value ^(1)^	*p*-Value ^(2)^
	**Baseline**	**Week 12**		**Baseline**	**Week 12**		
**Per protocol**							
***Actinomycetota***							
*Bifidobacterium adolescentis*	0.046 ± 0.076	0.070 ± 0.102	0.041 *	0.021 ± 0.039	0.040 ± 0.072	0.182	0.393
***Bacillota***							
*Anaerobutyricum soehngenii*	0.000 ± 0.000	0.000 ± 0.000	0.317	0.000 ± 0.000	0.000 ± 0.000	1.000	0.496
*Blautia faecicola*	0.002 ± 0.004	0.003 ± 0.005	0.505	0.001 ± 0.002	0.002 ± 0.004	0.038 *	0.845
*Coprococcus catus*	0.002 ± 0.001	0.002 ± 0.001	0.355	0.002 ± 0.001	0.002 ± 0.002	0.037 *	0.040 ^†^
*Coprococcus comes*	0.000 ± 0.000	0.000 ± 0.000	0.792	0.000 ± 0.000	0.000 ± 0.000	0.317	0.792
*Dorea phocaeensis*	0.000 ± 0.000	0.000 ± 0.000	0.062	0.000 ± 0.000	0.000 ± 0.000	0.593	0.062
*Evtepia gabavorous*	0.000 ± 0.000	0.000 ± 0.000	0.458	0.002 ± 0.010	0.000 ± 0.000	0.461	0.458
*Faecalibacillus intestinalis*	0.001 ± 0.001	0.001 ± 0.002	0.350	0.001 ± 0.002	0.001 ± 0.001	0.158	0.350
*Hominisplanchenecus faecis*	0.000 ± 0.000	0.000 ± 0.000	0.250	0.000 ± 0.000	0.000 ± 0.000	0.336	0.250
*Lachnospira eligens*	0.004 ± 0.006	0.003 ± 0.010	0.039 *	0.004 ± 0.008	0.002 ± 0.002	0.528	0.073
*Latilactobacillus sakei*	0.000 ± 0.001	0.002 ± 0.005	0.008 *	0.002 ± 0.005	0.000 ± 0.001	0.347	0.122
*Roseburia inulinivorans*	0.005 ± 0.006	0.002 ± 0.002	0.035 *	0.002 ± 0.003	0.002 ± 0.003	0.570	0.711
*Ruminococcoides bili*	0.002 ± 0.006	0.003 ± 0.011	0.500	0.006 ± 0.013	0.010 ± 0.023	0.139	0.041 ^†^
*Ruminococcus bromii*	0.006 ± 0.008	0.010 ± 0.014	0.099	0.009 ± 0.013	0.016 ± 0.024	0.013 *	1.000
*Turicibacter bilis*	0.000 ± 0.000	0.003 ± 0.007	0.805	0.000 ± 0.000	0.003 ± 0.005	0.717	0.805
*Vescimonas fastidiosa*	0.002 ± 0.003	0.001 ± 0.001	0.021 *	0.002 ± 0.003	0.002 ± 0.006	0.575	0.288
***Bacteroidota***							
*Alistipes onderdonkii*	0.002 ± 0.009	0.001 ± 0.002	0.851	0.004 ± 0.007	0.002 ± 0.003	0.041 *	0.165
*Bacteroides fragilis*	0.000 ± 0.001	0.002 ± 0.008	0.878	0.002 ± 0.003	0.001 ± 0.001	0.051	0.038 ^†^
*Bacteroides stercoris*	0.003 ± 0.006	0.008 ± 0.016	0.182	0.002 ± 0.004	0.001 ± 0.002	0.013 *	0.039 ^†^
*Phocaeicola plebeius*	0.005 ± 0.008	0.015 ± 0.020	0.005 *	0.010 ± 0.019	0.015 ± 0.030	0.422	0.354
***Pseudomonadota***							
*Enterobacter cloacae*	0.000 ± 0.000	0.000 ± 0.000	0.176	0.000 ± 0.000	0.000 ± 0.000	0.465	0.176

Values are mean ± SD. KP, kefir postbiotics. ^(1)^ *p*-values for comparison between baseline and week 12 were evaluated using Wilcoxon test. ^(2)^ *p*-values for changes between KP and placebo groups from baseline to week 12 were evaluated using Mann–Whitney U-test. * *p* < 0.05, ^†^ *p* < 0.05.

**Table 5:** Correlation between gut microbiota and biomarkers.

Parameter	Species Level	
**Dominant grip strength**	*Anaerobutyricum soehngenii*	*Hominisplanchenecus faecis*
	0.69 (0.024)	−0.68 (0.028)
IL-1β	*Coprococcus catus*	*Evtepia gabavorous*
	0.72 (0.013)	0.67 (0.039)
IL-2	*Coprococcus comes*	
	−0.70 (0.022)	
Myostatin	*Dorea phocaeensis*	
	0.72 (0.014)	
Irisin	*Faecalibacillus intestinalis*	*Turicibacter bilis*
	0.66 (0.045)	−0.68 (0.032)

Values are mean ± SD. KP, kefir postbiotics; IL-1β, interleukin-1 beta; IL-2, interleukin-2; IGF-1, Insulin-like growth factor 1.

## Data Availability

The data presented in this study are available on request from the corresponding author due to ethical and privacy restrictions related to human participants.

## Supplementary Materials

The following supporting information can be downloaded at: https://www.mdpi.com/2072-6643/17/24/3937/s1. Table S1 presents physical activity (GPAQ) and dietary intake of participants; Table S2 summarizes blood biomarkers, Table S3 reports vital signs, all assessed during the 12-week intervention period.
