# Supplementary material for: Bioconversion-Based Postbiotics Enhance Muscle Strength and Modulate Gut Microbiota in Healthy Individuals: A Randomized, Double-Blind, Placebo-Controlled Trial
Source: Nutrients. 2025 Dec 17;17(24):3937. doi: 10.3390/nu17243937 (PMC12813636; doi:10.3390/nu17243937)
Supplement: Supplementary file 1 [file nutrients-17-03937-s001.zip › nutrients-4024797-supplementary.pdf]

Supplementary Materials

Table S1. Physical activity and food intake of participants for 12 weeks

	KP		Placebo		*P*-value
	Baseline	Week 12	Baseline	Week 12	
**Intention to treat**	(n = 27)		(n = 26)		
GPAQ (MET-min/week)	796.30 ± 1167.25	796.30 ± 1240.89	426.92 ± 438.87	445.38 ± 351.43	0.728
Dietary intake					
Energy intake (kcal/day)	1501.05 ± 663.36	1610.28 ± 470.14	1452.92 ± 343.57	1374.10 ± 420.42	0.057
Carbohydrate (g)	225.17 ± 105.28	238.65 ± 75.28	214.50 ± 59.15	201.94 ± 80.40	0.067
Fat (g)	38.81 ± 22.32	44.21 ± 22.66	37.02 ± 15.57	35.86 ± 17.78	0.328
Protein (g)	61.76 ± 23.46	62.72 ± 21.02	63.87 ± 22.90	56.35 ± 19.31	0.220
Total fiber (g)	18.59 ± 9.19	19.68 ± 7.30	16.90 ± 5.05	14.57 ± 6.01	0.054
**Per protocol**	(n = 22)		(n = 23)		
GPAQ (MET-min/week)	822.73 ± 1273.92	826.36 ± 1346.82	438.26 ± 458.69	422.61 ± 360.97	0.439
Dietary intake					
Energy intake (kcal/day)	1504.94 ± 716.93	1629.95 ± 505.28	1420.86 ± 346.01	1358.04 ± 445.28	0.080
Carbohydrate (g)	226.55 ± 113.76	242.50 ± 82.49	209.86 ± 61.13	198.77 ± 84.46	0.059
Fat (g)	39.54 ± 24.33	45.13 ± 24.06	36.17 ± 16.15	35.57 ± 18.71	0.525
Protein (g)	59.39 ± 24.25	60.66 ± 21.35	62.74 ± 23.42	56.10 ± 20.47	0.307
Total fiber (g)	19.01 ± 9.74	19.84 ± 7.34	16.19 ± 4.64	14.51 ± 6.37	0.233

KP, kefir postbiotics; GPAQ, Global Physical Acid Quetionnaire.

1) P-values for differences between KP and placebo groups at baseline were determined by the independent t-test ($P < 0.05$). * indicates a significant difference between KP and Placebo ($P < 0.05$).

Table S2. Blood biomarkers of participants for 12 weeks

	KP		Placebo		*P*-value	
	Baseline	Week 12	Baseline	Week 12	Baseline	Week 12
**Intention to treat**	(n = 27)		(n = 26)			
WBC (10^3/μL)	4.66 ± 1.24	4.77 ± 1.11	5.02 ± 1.29	5.00 ± 1.06	0.301	0.193
RBC (10^6/μL)	4.33 ± 0.40	4.31 ± 0.44	4.27 ± 0.34	4.22 ± 0.33	0.535	0.412
Hemoglobin (g/dL)	13.09 ± 1.41	13.04 ± 1.30	13.13 ± 1.00	12.96 ± 0.90	0.769	0.533
Hematocrit (%)	39.27 ± 3.81	39.04 ± 3.54	39.50 ± 2.87	38.84 ± 2.58	0.809	0.821
Platelet (10^3/μL)	266.59 ± 53.33	265.89 ± 55.98	262.69 ± 41.02	253.50 ± 43.44	0.767	0.374
Seg neutrophil (%)	53.41 ± 7.16	55.20 ± 8.64	55.24 ± 9.78	56.18 ± 8.45	0.441	0.677
Lymphocyte (%)	36.25 ± 6.91	35.01 ± 8.21	33.79 ± 8.72	33.52 ± 8.32	0.260	0.515
Monocyte (%)	7.09 ± 1.39	6.88 ± 1.56	7.21 ± 1.93	6.96 ± 1.64	0.804	0.856
Eosinophil (%)	2.57 ± 1.78	2.21 ± 1.26	3.00 ± 2.18	2.55 ± 2.05	0.471	0.637
Basophil (%)	0.67 ± 0.25	0.70 ± 0.33	0.77 ± 0.31	0.78 ± 0.32	0.345	0.254
Protein, total (g/dL)	7.38 ± 0.35	7.34 ± 0.37	7.47 ± 0.40	7.33 ± 0.43	0.400	0.982
Albumin (g/dL)	4.39 ± 0.27	4.41 ± 0.22	4.31 ± 0.19	4.31 ± 0.24	0.187	0.133
Calcium (mg/dL)	9.24 ± 0.33	9.18 ± 0.27	9.07 ± 0.31	9.01 ± 0.41	0.052	0.083
Phosphorus (mg/dL)	3.49 ± 0.45	3.57 ± 0.55	3.47 ± 0.46	3.53 ± 0.31	0.993	0.747
Glucose (mg/dL)	99.96 ± 6.38	100.04 ± 7.62	102.88 ± 7.98	101.85 ± 7.57	0.392	0.605
BUN (mg/dL)	13.16 ± 3.53	13.65 ± 3.97	13.73 ± 3.91	13.84 ± 3.74	0.581	0.856
Creatinine (mg/dL)	0.66 ± 0.10	0.63 ± 0.12	0.66 ± 0.14	0.63 ± 0.15	0.695	0.859
Bilirubin, total (mg/dL)	0.77 ± 0.26	0.65 ± 0.15	0.78 ± 0.27	0.77 ± 0.33	0.943	0.328

ALP (U/L)	67.04 ± 17.15	67.96 ± 16.49	65.88 ± 17.93	62.00 ± 14.47	0.812	0.169
AST (U/L)	27.89 ± 6.33	27.26 ± 7.15	28.15 ± 6.34	27.62 ± 6.28	0.735	0.761
ALT (U/L)	18.15 ± 8.57	16.96 ± 7.43	15.73 ± 9.06	15.65 ± 8.84	0.199	0.281
Hemoglobin A1c (%)	0.07 ± 0.07	0.09 ± 0.10	0.08 ± 0.07	0.08 ± 0.08	0.064	0.741
CRP (mg/L)	0.05 ± 0.01	0.06 ± 0.02	0.06 ± 0.02	0.05 ± 0.01	0.900	0.844
Cholesterol (mg/dL)	210.74 ± 39.65	201.70 ± 39.63	198.08 ± 42.37	197.77 ± 37.63	0.266	0.713
Triglyceride (mg/dL)	96.33 ± 40.05	101.00 ± 46.83	101.00 ± 72.13	99.88 ± 66.22	0.505	0.350
HDL-C (mg/dL)	60.93 ± 10.19	56.93 ± 10.49	124.70 ± 36.94	118.48± 38.70	0.866	0.332
LDL-C (mg/dL)	124.70 ± 36.94	118.48 ± 38.70	110.85 ± 37.33	110.88 ± 37.93	0.180	0.474
**Per protocol**	(n = 22)		(n = 23)			
WBC (10^3/μL)	4.72 ± 1.19	4.75 ± 1.11	5.05 ± 1.37	5.03 ± 1.12	0.453	0.224
RBC (10^6/μL)	4.28 ± 0.42	4.26 ± 0.47	4.25 ± 0.35	4.21 ± 0.35	0.822	0.666
Hemoglobin (g/dL)	12.96 ± 1.53	12.92 ± 1.40	13.10 ± 1.05	12.95 ± 0.94	1.000	0.785
Hematocrit (%)	38.89 ± 4.09	38.75 ± 3.84	39.37 ± 2.98	38.75 ± 2.67	0.654	0.995
Platelet (10^3/μL)	274.55 ± 50.15	273.68 ± 53.61	265.96 ± 41.76	256.87 ± 43.32	0.535	0.253
Seg neutrophil (%)	53.34 ± 7.33	55.08 ± 8.95	55.76 ± 10.26	56.20 ± 9.00	0.369	0.679
Lymphocyte (%)	36.47 ± 6.95	35.32 ± 8.36	33.45 ± 9.19	33.60 ± 8.84	0.222	0.506
Monocyte (%)	7.18 ± 1.44	6.84 ± 1.68	6.98 ± 1.79	6.77 ± 1.45	0.677	0.887
Eosinophil (%)	2.34 ± 1.82	2.03 ± 1.26	3.03 ± 2.31	2.63 ± 2.16	0.246	0.312
Basophil (%)	0.67 ± 0.26	0.78 ± 0.32	0.73 ± 0.35	0.81 ± 0.33	0.336	0.348
Protein, total (g/dL)	7.34 ± 0.32	7.30 ± 0.35	7.48 ± 0.42	7.34 ± 0.45	0.210	0.749
Albumin (g/dL)	4.35 ± 0.25	4.38 ± 0.22	4.31 ± 0.20	4.30 ± 0.25	0.545	0.284
Calcium (mg/dL)	9.20 ± 0.34	9.06 ± 0.32	9.14 ± 0.28	9.01 ± 0.42	0.154	0.216

Phosphorus (mg/dL)	3.43 ± 0.44	3.45 ± 0.49	3.52 ± 0.56	3.52 ± 0.33	0.918	0.431
Glucose (mg/dL)	99.95 ± 6.24	100.55 ± 7.96	103.17 ± 8.36	102.35 ± 7.84	0.152	0.448
BUN (mg/dL)	12.45 ± 3.06	13.26 ± 4.25	13.89 ± 4.07	13.73 ± 3.94	0.188	0.701
Creatinine (mg/dL)	0.66 ± 0.11	0.63 ± 0.13	0.67 ± 0.15	0.64 ± 0.15	0.919	0.919
Bilirubin, total (mg/dL)	0.78 ± 0.28	0.65 ± 0.16	0.80 ± 0.27	0.78 ± 0.35	0.708	0.358
ALP (U/L)	64.59 ± 16.63	64.64 ± 15.45	66.30 ± 19.05	61.87 ± 1.54	0.750	0.539
AST (U/L)	26.68 ± 4.84	26.14 ± 5.99	27.91 ± 6.36	27.48 ± 6.51	0.524	0.569
ALT (U/L)	16.05 ± 6.85	15.68 ± 6.92	16.22 ± 9.49	15.87 ± 9.07	0.802	0.649
Hemoglobin A1c (%)	0.06 ± 0.05	0.08 ± 0.09	0.09 ± 0.08	0.08 ± 0.09	0.036*	0.584
CRP (mg/L)	0.05 ± 0.01	0.06 ± 0.02	0.06 ± 0.02	0.05 ± 0.01	0.802	0.802
Cholesterol (mg/dL)	213.86 ± 42.68	204.82 ± 42.50	199.70 ± 44.08	200.52 ± 38.09	0.334	0.650
Triglyceride (mg/dL)	98.36 ± 42.70	103.00 ± 50.78	105.09 ± 75.51	104.35 ± 68.54	0.658	0.532
HDL-C (mg/dL)	60.95 ± 10.22	58.13 ± 13.53	59.57 ± 13.28	58.13 ± 13.53	0.201	0.533
LDL-C (mg/dL)	127.95 ± 39.31	121.45 ± 41.06	112.78 ± 38.99	114.00 ± 37.56	0.697	0.703

Values are means ±SDs.
KP, Kefir postbiotics; WBC, White blood cells; RBC, Red blood cells; ALP, Alkaline phosphatase; AST, Aspartate aminotransferase; ALT, Alanine aminotransferase; HDL, High density lipoprotein; LDL, Low density lipoprotein.
P-values for differences between KP and placebo groups were determined by the independent *t*-test. ($P < 0.05$) * indicates a significant difference between KP and Placebo ($P<0.05$).

Table S3. Vital signs of participants for 12 weeks.

	KP		Placebo		*P*-value	
	Baseline	Week 12	Baseline	Week 12	Baseline	Week 12
**Intention to treat**	(n = 27)		(n = 26)			
SBP(mmHg)	118.22 ± 18.92	115.81 ± 15.58	117.85 ± 13.63	112.27 ± 12.28	0.934	0.371
DBP(mmHg)	69.11 ± 12.28	68.33 ± 10.46	67.73 ± 9.49	66.85 ± 8.07	0.972	0.755
Pulse rate(beats/min)	76.70 ± 9.81	75.37 ± 10.97	76.65 ± 10.81	74.92 ± 12.85	0.796	0.845
Body temperature(°C)	36.51 ± 0.22	36.54 ± 0.20	36.59 ±0.24	36.56 ± 0.15	0.207	0.971
**Per protocol**	(n = 22)		(n = 23)			
SBP(mmHg)	119.82 ± 19.31	116.14 ± 1.25	111.70 ± 12.46	117.61 ± 12.71	0.651	0.308
DBP(mmHg)	71.05 ± 12.67	69.68 ± 10.67	67.57 ± 8.79	66.52 ± 7.37	0.510	0.306
Pulse rate(beats/min)	76.95 ± 10.11	75.41 ± 11.96	76.70 ± 10.73	74.96 ± 12.70	0.794	0.847
Body temperature(°C)	36.50 ± 0.23	36.55 ± 0.18	36.61 ± 0.24	36.53 ± 0.12	0.148	0.470

Values are means ±SDs.

KP, kefir postbiotics; SBP, Systoloic blood pressure; DBP, Diastolic blood pressure.

P-values for differences between KP and placebo groups were determined by the independent *t*-test. ($P < 0.05$) * indicates a significant difference between KP and Placebo (P<0.05).
